# Delayed acyclovir therapy for disseminated varicella zoster in an adult kidney transplant recipient: a case report and literature review

**DOI:** 10.1097/MS9.0000000000000231

**Published:** 2023-03-02

**Authors:** Mohammad Alsultan, Marwa Kliea, Qussai Hassan, Kassem Basha

**Affiliations:** a Department of Nephrology, Al Assad and Al Mouwasat University Hospitals, Damascus University Faculty of Medicine; b Department of Neurology, Al Assad and Al Mouwasat University Hospitals, Damascus University Faculty of Medicine; c Nephrology Department, Al Assad University Hospital, Damascus University Faculty of Medicine; d Nephrology Department, Al Mouwasat University Hospitals, Damascus University Faculty of Medicine, Damascus, Syria

**Keywords:** acyclovir (ACV), kidney transplant, pneumonitis, Syria, varicella-zoster virus (VZV)

## Abstract

**Introduction:**

Kidney transplant recipients are at increasing risk for reactivation of varicella-zoster virus (VZV) infection.

**Presentation of case:**

A 31-year-old male was admitted with fever, chest pain, and dyspnea. Also, the complaints accompanied by generalized maculopapular, vesicular, hemorrhagic, itching, and painful rash with pustules and crusts on an erythematous base fill the entire body for the last 10 days. Chest computed tomography scan showed diffuse miliary and ground-glass opacities. The patient had a previous history of chickenpox infection in childhood, no recent contact with individuals suffering from VZV infection, and no known pretransplant serology for VZV. Due to the high clinical suspicion of reactivated VZV with pneumonitis and severe disseminated form, we started the treatment with intravenous acyclovir (ACV) for 10 days followed by oral ACV for a total of 21 days, along with stopping mycophenolate mofetil and increasing the prednisolone dose to 10 mg/d. The clinical status was improved and the rash receded with a flaked surface for old lesions.

**Conclusion:**

We experienced a successful ACV treatment for delayed and severe VZV infection with a literature review of VZV pneumonitis among kidney transplant recipients. To the best of our knowledge, this is the first case that presented a disseminated skin form with pneumonitis of VZV from Syria. This case supports the initiation of antiviral therapy for transplant patients even after 72 hours the onset of the rash despite the lack of evidence in these circumstances.

Highlights

- Severe and disseminated varicella-zoster virus (VZV) infection with diffuse pulmonary involvement, was resolved by acyclovir (ACV).
- Radiologic and clinical findings helping in VZV diagnosis without serologic tests.
- Empiric antiviral therapy should be started as soon as possible and can be lifesaving.
- This case supports the initiation of antiviral for transplant patients even later than 72 hours of the rash.

## Introduction

Immunosuppressive agents have dramatically reduced the incidence of rejection of transplanted organs while increasing patients’ susceptibility to opportunistic bacterial infections, in addition, to be susceptible to a broad array of viral pathogens^1^,^2^. Kidney transplant recipients are at increased risk of developing viral infections that are a result of community exposures, transmission from the allograft, and sometimes, viruses like VZV can be reactivated in the setting of immunosuppression^2^.

VZV is one of eight herpes viruses known to cause two clinically distinct forms of the disease: varicella (chickenpox); which results from primary VZV infection, and herpes zoster (HZ) (shingles); which results from endogenous reactivation of latent VZV^3^. Immunocompromised populations have a reduction in the immune response that could lead to the reactivation of VZV from latent infection in the sensory ganglia^4^. The incidence of VZV in solid organ transplant (SOT) recipients is 10-fold to 100-fold higher than the general population, especially during the first 4 years posttransplant^5^,^6^. The type of transplantation, immunosuppression and antiviral prophylaxis may influence HZ incidence. According to several trials and retrospective studies published in the last decade, the incidence of HZ varied between 3.5 and 9% in kidney transplant recipients^7^,^8^. Dissemination similar to that seen in primary VZV infection is uncommon but has been reported in SOT and other immunocompromised populations^6^. More severe complications such as disseminated zoster occur mainly in immunocompromised patients known as disseminated HZ, which is characterized by vesicles spreading beyond the affected dermatome and potentially affecting organs other than the skin that leading to pneumonia, encephalitis, pancreatitis, and hepatitis^9^.

Here, we present a reactivated VZV infection in a kidney transplant patient who presented with severe disseminated skin lesions accompanied by pneumonia.

## Presentation of case

This case report is according with the SCARE guidelines and written informed consent was obtained from the patient for publication of this case report and accompanying images^10^.

A 31-year-old male was admitted to our emergency department with fever, chest pain, dyspnea, diarrhea, and generalized rash. The sequential of symptoms were as follows: a large volume of watery diarrhea (two to three times a day) with generalized abdominal pain started from the past month, followed – in the last 10 days – by malaise, fever (two to three times a day), chest pain, and progressive dyspnea (New York Heart Association functional class II). The latter complaints were accompanied by an itching and painful rash that arose on the head and then generalized including the whole body.

The patient’s medical history comprised of chronic kidney disease of unknown origin followed by kidney transplantation (living donor) from 6 years. Currently, he had chronic kidney disease on a transplanted kidney with basal creatinine levels (4.5–5 mg/dl) and urinary output of ∼2 l/d.

Also, the patient had a previous admission – in the past 3 months – for studying chronic watery diarrhea (three to four times a day) with considerable weight loss (about 20 kg). At this point, the patient was diagnosed and treated for chronic *Clostridium difficile* infection with positive fecal toxins (A and B), when upper and lower gastrointestinal endoscopies with biopsies and the whole body CT scans were negative for malignancies. His medications were atorvastatin 10 mg/d, prednisolone 5 mg/d, tacrolimus 1 mg/b.i.d., and mycophenolate mofetil 500 mg/b.i.d.

Physical examination on admission was; blood pressure 80/50 mmHg, respiratory rate 24/min, heart rate 114 beats/min, temperature 39°C, oxygen saturation 96% in air room, fine crackles in chest auscultation, epigastric tenderness, and urinary output reduced to 0.5 l/d.

**Figure 1:**
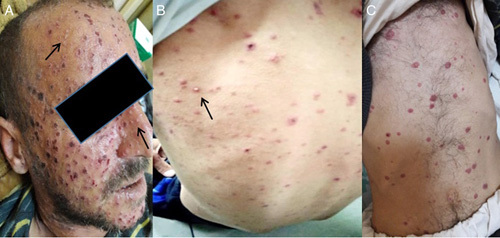
(A) Maculopapular (arrows) rash with hemorrhagic crusts on an erythematous base on the face. Different stages of development at the same time. (B) Maculopapular, vesicular, and hemorrhagic rash with pustules (arrow) on the back. (C) Hemorrhagic rash on the abdomen.

**Figure 2:**
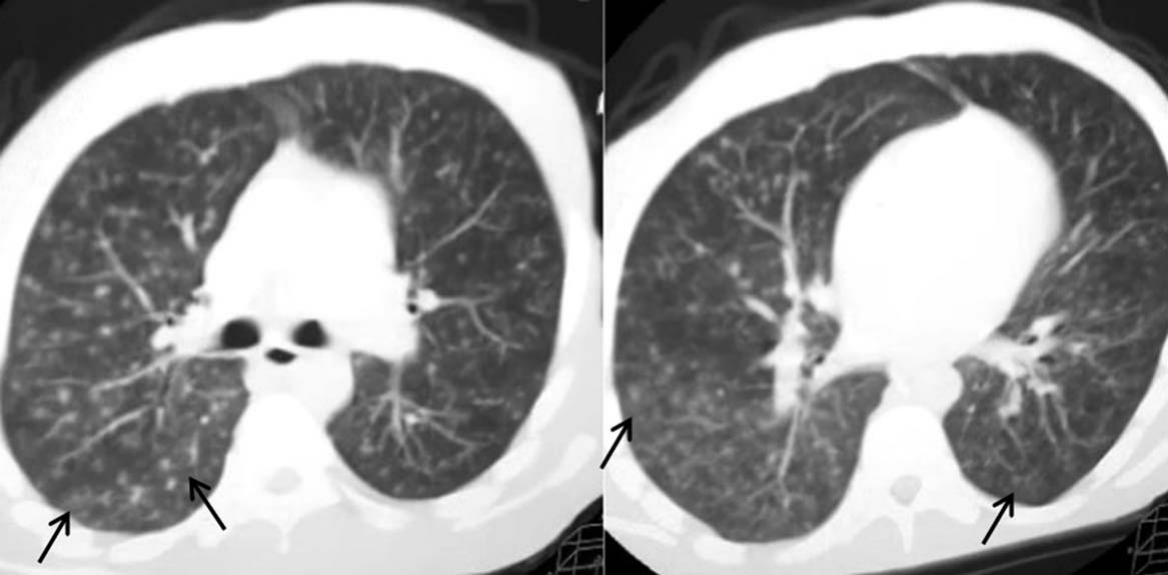
Chest computed tomography; diffuse miliary and ground-glass opacities (arrows).

Also, a maculopapular, vesicular, and hemorrhagic rash with pustules and crusts on an erythematous base fills the entire body. The rash predominantly showed on the face and the trunk (Fig. 1). Laboratory tests on admission are shown in Table 1. ECG showed sinus tachycardia. Chest and abdominal CT scans (Fig. 2) showed diffuse nodules opacities and dilation with thickening walls of intestinal loops.

**Table 1:** Laboratory tests on admission

WBC	7.2	TP	5	CRP	2.2
N%	89%	ALB	2.9	LDH	245
L%	15%	AST	31	PTH	162
HB	13.9	ALT	16	pH	7.16
HT	41	TB	0.28	HCO_3_	6.8
PLT	152	Na	127	PO_2_	121
Ur	203	K	2.7	PCO_2_	18
Cr	7.4	Ca	7.3	SO_2_%	97%
Glu	87	ESR	20	Toxin A, B	Negative
Additional					
TST	Negative	Gene expert	Negative	AFB in 3 consecutive sputum test	Negative

AFB, acid-fast bacilli; ALB, albumin; Ca, calcium; Cr, creatinine; CRP, C-reactive protein (up to 0.5 mg/dl); ESR, erythrocyte sedimentation rate; Glu, glucose; HB, hemoglobin; HT, hematocrit; L%; lymphocytes percentage; LDH, lactate dehydrogenase; N%, neutrophils percentage; PLT, platelets; PTH, parathyroid hormone; Toxin A, B; fecal toxins for *Clostridium difficile*; TP, total protein; TST, tuberculin skin test; Ur, urea; WBC, white blood count.

The treatment consisted of fluids, electrolytes replacement, and managing the acidosis with sodium bicarbonate. Due to the previous history of *C. difficile*, we started the treatment with oral vancomycin (125 mg/four times daily) with subsequent tapering for recurrent cases of *C. difficile* infection^11^.

Also, due to the first and high clinical suspicion by a conjunction of skin appearance and radiologic findings of pneumonitis, we started the treatment for VZV with intravenous ACV 10 mg/kg/d (600 mg/d) along with stopping mycophenolate mofetil and increasing the prednisolone dose to 10 mg/d. Additional tests were obtained to rule out miliary tuberculosis (additional Table 1).

The patient had a previous history of chickenpox infection in childhood, no recent contact with individuals suffering from VZV infection, and no known pretransplant serology for VZV. In addition, there was no other neurological, hepatic, ocular, or pancreatic complications.

**Figure 3:**
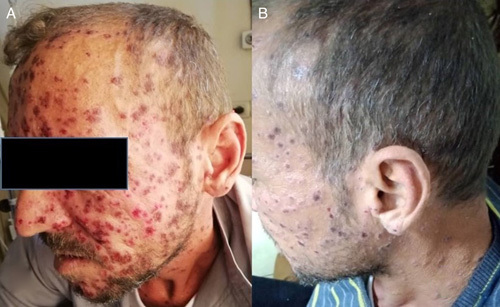
(A) Crusts on a hemorrhagic base for old lesions without new vesicles (day 7 after starting the acyclovir). (B) Crusts without erythematous base (healing phase), day 10 of treatment.

The clinical status was improved, where diarrhea resolved, fever disappeared after 2 days, dyspnea resolved after 4 days, and urinary output improved ∼to 4 to 5 l/d. The rash receded and did not show new vesicles with a flaked surface for old lesions (Fig. 3). The serum creatinine rose during intravenous ACV administration, which reached 8 mg/dl, despite adequate fluid administration but returned to the basal range after completing the intravenous treatment for 10 days. Thereafter oral ACV was continued to complete a total 21 days course.

## Discussion

We experienced a case of VZV infection in a kidney transplant patient, in which severe and disseminated VZV infection with diffuse pulmonary involvement, was successfully and fortunately resolved by ACV. To the best of our knowledge, this is the first case that presented a disseminated skin form with pneumonitis of VZV from Syria.

Diagnosis of HZ in transplant recipients is often difficult, because mucocutaneous involvement may present with atypical forms that could mimic other diseases, such as herpes simplex virus infection, and may present with multiorgan involvement^4^. Also, cutaneous lesions eventually develop but their appearance may be delayed and atypical because of hemorrhagic appearance^5^. Visceral dissemination is a rare and life-threatening emergency that may present as a fulminant and rapidly evolving syndrome and may occasionally develop in the absence of a rash^12^–^14^.

Our patient presented with a severe and hemorrhagic cutaneous form of VZV simultaneously with pneumonitis. In general, both primary varicella and HZ have typical clinical presentations that allow for a presumptive clinical diagnosis^6^. Although laboratory testing in SOT recipients is more important, including PCR which is the method of choice and the most sensitive test for VZV diagnosis; because a diagnosis may be more difficult to establish on clinical grounds alone^6^.

Also, the vast majority of adults (at least 95%) had a prior VZV infection. Thus, in adult transplant recipients, VZV disease is predominantly due to reactivation rather than primary infection although rare cases of primary infection have been reported^5^.

In our patient, a previous history of chickenpox in childhood and no recent contact with individuals suffering from varicella infection. This seems most likely due to reactivated VZV infection. However PCR did not available during admission in our condition, the skin lesions with different stages of development at the same time induced the clinical suspicion of VZV diagnosis. In addition, a prompt and high suspicion of the diagnosis with the empiric institution of antiviral therapy resulted in resolving the disease.

VZV pneumonitis in transplant recipients has been associated with high mortality (10–33%) despite the prompt diagnosis and the empiric institution of antiviral therapy^13^–^15^. A multicenter cohort study reported VZV-related community-acquired pneumonia in 29 ICUs from 1996 to 2015. A total of 102 patients were encountered and 53 patients (52%) were immunocompromised. Overall ICU and hospital mortality were 17 and 24%, respectively^16^.

There is a lack of trials evaluating the initiation of a systemic antiviral medication more than 72 hours after the onset of the rash due to there is likely minimal benefit. However, there is no evidence basis to recommend the administration of antivirals in this setting, based on consensus and as recommended in guidelines, all immunocompromised patients should be initiated antivirals even if they present after 72 hours^17^. This is particularly critical in the SOT recipient^17^.

Here, we reported a successful ACV treatment for delayed and severe VZV infection (10 days after the onset of the rash), that was accompanied by disseminated and visceral disease. This case supports the initiation of antiviral therapy for transplant patients even later than 72 hours of skin rash.

A literature review for pneumonitis in kidney transplant recipients (Table 2) showed only four published articles^1^,^18^–^20^. Another two retrospective studies were excluded due to a lack of descriptive data on VZV pneumonitis^16^,^21^.

**Table 2:** Literature review for VZV pneumonia in adult with renal transplant

References	Age/sex	Donor living/deceased	Serostatus of VZV-IgG before transplantation	Time from transplant	Diagnostic method of pneumonia (CT/others)	Skin distribution/other organ involvement	Immunosuppressant	Treatment/onset from symptoms	Outcome
Takahashi *et al.* 18	50/male	Living	Positive	2 years	CT (bilateral GGO and bilateral multiple patchy ground glass)/sputum positive PCR	Vesicles (ramsay hunt)/CNS symptoms	Prednisolone/TAC/MMF	ACV/11 days meropenem, levofloxacin, methylprednisolone	Healing
Helou *et al.* 19	49/female	Living	NA	4 years	BALF VZV positive PCR	CNS symptoms/vesicles on mucosa and supraglottic swelling	Prednisolone/belatacept/MMF	Vancomycin, piperacillin–tazobactam, fluconazole. CS inhalation ACV/23 days	Died
Milinkovic *et al.* 1 (cohort study, the fifth patient)	36/male	Deceased	Positive	7 months	Chest radiography (interstitial pneumonia)/BALF VZV positive PCR	Rash on left inguinal and sacral region	Prednisolone/TAC/MMF	GCV/2 days, ACV/12 days, methylprednisolone (for 3 days)	Died
Rodriguez-Moreno *et al.* 20 (cohort study, first, second, and eighth patients)	58	NA	Negative	Median interval 32 months	Clinical symptoms/IgG-IgM conversion/VZV PCR	Vesicles	Prednisolone/CyA/MMF	ACV+GCV	Healing
	64		Positive			Vesicles	Prednisolone/TAC/MMF	ACV+IVIG	Healing

ACV, acyclovir; BALF, bronchoalveolar fluid; CNS, central nervous system; CS, corticosteroids; CyA, cyclosporine; DIC, disseminated intravascular coagulation; F, female; GCV, ganciclovir; GGO, ground-glass opacities; Ig, immunoglobulin; IVIG, intravenous immunoglobulin; M, male; MMF, mycophenolate mofetil; NA, not available; TAC, tacrolimus; VZV, varicella zoster virus.

The first was a 10 years study from India, that reported 23 patients infected with VZV^21^. Of them, 30.4% of the patients had presented with infections in the form of pneumonitis, and mortality was reported in three patients (8.6%)^21^. The second was a retrospective multicenter cohort study in 29 French ICUs^16^. This study reported VZV pneumonia in 11 patients of SOT but did not define the transplanted organ^16^.

The included studies were three case reports and one cohort study, that concluded five patients^1^,^18^–^20^. The skin rash mentioned in cases^1^,^18^,^19^, was limited and the fourth cohort study did not define the skin distribution^20^. Radiologic findings were reported in two cases, which consisted of interstitial lesions or ground-glass opacities (GGO)^1^,^18^. Two patients experienced central nervous system involvement in addition to skin and pulmonary involvements^18^,^19^. All patients received antiviral therapy, predominantly ACV, however, two of five patients died^1^,^19^.

Radiologic findings of VZV on CT scan usually show 1–10 mm well-defined or ill-defined nodules with a surrounding halo of GGO, patchy GGO, and coalescence of nodules diffusely throughout both lungs^22^,^23^. A miliary distribution may also occur^22^,^23^. These small nodules also can be seen in patients with other diseases such as pulmonary tuberculosis or pneumoconiosis, however, the diagnosis of VZV infection usually can be established on the basis of clinical findings^22^,^23^.

In our patient, the radiologic finding (a miliary form and GGO) aligns with clinical findings (hemorrhagic visculopapular rash) helping in VZV diagnosis without serologic tests. In general practice, many times the empiric treatment should be started as soon as possible and can be lifesaving such as in this case, where the empiric antiviral therapy achieved the desired goal^24^.

## Conclusion

We experienced a successful ACV treatment for delayed and severe VZV infection, that presented with disseminated skin lesions and pneumonitis. A prompt an empiric antiviral therapy should be start as soon as possible and can be lifesaving. However, this case supports the initiation of an antiviral therapy for delayed VZV infection in transplant patients, further studies are needed to prove the benefit of this late antiviral administration.

## Ethical approval

Written informed consent was obtained from the patient for publication of this case report and accompanying images, in line with local ethical approval requirements and in accordance with the Helsinki declaration.

## Consent

Written informed consent was obtained from the patient for publication of this case report and accompanying images. A copy of the written consent is available for review by the Editor-in-Chief of this journal on request.

## Sources of funding

This research did not receive any specific grant from funding agencies in the public, commercial, or not-for-profit sectors.

## Authors’ contribution

M.A. wrote the manuscript, literature search, submitted the article, and patient follow-up. M.K. makes case presentation section, literature search, and article corrections. Q.H. makes article corrections, supervision, and follow-up of the patient. K.B. makes article corrections, supervision, and follow-up of the patient.

## Conflicts of interest disclosure

The authors declare that they have no financial conflict of interest with regard to the content of this report.

## Research registration unique identifying number (UIN)

None.

## Guarantor

Mohammad Alsultan.

## Provenance and peer review

Not commissioned, externally peer-reviewed.
